# Clinical Utility of Thigh and Mid‐Thigh Dual‐Energy x‐Ray Absorptiometry to Identify Bone and Muscle Loss

**DOI:** 10.1002/jbm4.10704

**Published:** 2023-02-16

**Authors:** Sara Vogrin, Ebrahim Bani Hassan, Fernan Munandar Putra, Julie A Pasco, Mark A Kotowicz, Gustavo Duque

**Affiliations:** ^1^ Australian Institute for Musculoskeletal Science (AIMSS), Geroscience & Osteosarcopenia Research Program The University of Melbourne and Western Health St. Albans Victoria Australia; ^2^ Department of Medicine‐Western Health The University of Melbourne St. Albans Victoria Australia; ^3^ Melbourne Biotechnology Co Ltd. Greensborough Victoria Australia; ^4^ IMPACT‐Institute for Mental and Physical Health and Clinical Translation, School of Medicine Deakin University Geelong Victoria Australia; ^5^ Barwon Health University Hospital Geelong Geelong Victoria Australia; ^6^ Research Institute of the McGill University Health Centre Montreal Quebec Canada

**Keywords:** dual‐energy X‐ray absorptiometry (DXA), lean mass, mid‐thigh, osteoporosis, osteosarcopenia

## Abstract

Sarcopenia and osteoporosis are highly prevalent syndromes in older people, characterized by loss of muscle and bone tissue, and related to adverse outcomes. Previous reports indicate mid‐thigh dual‐energy X‐ray absorptiometry (DXA) is well suited for the simultaneous assessment of bone, muscle, and fat mass in a single scan. Using cross‐sectional clinical data and whole‐body DXA images of 1322 community‐dwelling adults from the Geelong Osteoporosis Study (57% women, median age 59 years), bone and lean mass were quantified in three unconventional regions of interest (ROIs): (i) a 2.6‐cm‐thick slice of mid‐thigh, (ii) a 13‐cm‐thick slice of mid‐thigh, and (iii) the whole thigh. Conventional indices of tissue mass were also calculated (appendicular lean mass [ALM] and bone mineral density [BMD] of lumbar spine, hip, and femoral neck). The performance of thigh ROIs in identifying osteoporosis, osteopenia, low lean mass and strength, past falls, and fractures was evaluated. All thigh regions (especially whole thigh) performed well in identifying osteoporosis (area under the receiver‐operating characteristic [ROC] curve [AUC] > 0.8) and low lean mass (AUC >0.95), but they performed worse in the diagnosis of osteopenia (AUC 0.7–0.8). All thigh regions were equivalent to ALM in discrimination of poor handgrip strength, gait speed, past falls, and fractures. BMD in conventional regions was more strongly associated with past fractures than thigh ROIs. In addition to being faster and easier to quantify, mid‐thigh tissue masses can be used for identifying osteoporosis and low lean mass. They are also equivalent to conventional ROIs in their associations with muscle performance, past falls, and fractures; however, further validation is required for the prediction of fractures. © 2022 The Authors. *JBMR Plus* published by Wiley Periodicals LLC on behalf of American Society for Bone and Mineral Research.

## Graphical abstract

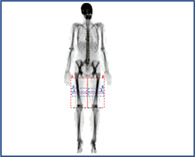

## Introduction

The aging population is expanding worldwide and is associated with a higher prevalence of chronic diseases. Hence, conditions affecting body composition, such as sarcopenia, osteoporosis, osteosarcopenia, cachexia, eating disorders, frailty, obesity, and their associated adverse outcomes, are on the rise.^(^ ^1^, ^2^, ^3^ ^)^ Bone, skeletal muscle, and fat are closely interconnected, not only anatomically but also mechanically, metabolically, and chemically.^(^ ^4^ ^)^ Furthermore, pathophysiological and epidemiological research indicates that the decline of bone or muscle can impact the volume of the other, with both tissues being replaced with ectopic fat that plays an active role in their deterioration.^(^ ^5^ ^)^ In addition, nearly two‐thirds of older inpatients, up to 80% of nursing home residents, and around 80% of community‐dwelling older adults have at least one of the aforementioned exemplified syndromes.^(^ ^6^, ^7^ ^)^ Overall, the impact of these conditions on health is an increased risk of frailty, disability, and adverse outcomes.^(^ ^8^ ^)^

The concomitant decline of bone and muscle, with or without changes in fat mass quantity and distribution, is not limited to older adults. Within just 48 hours, patients admitted to an intensive care unit (ICU) could lose 1 kg of muscle mass and 2% of bone mass.^(^ ^9^ ^)^ Furthermore, major changes in body composition and their associated outcomes are still a significant concern in patients with eating disorders,^(^ ^10^ ^)^ malignancies,^(^ ^11^ ^)^ and other acute or chronic conditions.^(^ ^12^ ^)^ In hospital inpatients of all ages, these conditions predict prolonged stay, poor functional recovery after hospitalization, and resistance to interventions (i.e., nutrition and exercise).^(^ ^13^, ^14^, ^15^ ^)^ Hence, timely, affordable, and rapid assessment of muscle, bone, and fat in a wide range of conditions is desirable.

The diagnosis of these syndromes usually involves the use of imaging. Depending on which definition of sarcopenia is chosen, assessment includes muscle strength and physical performance with or without muscle mass (or lean mass as measured by dual‐energy X‐ray absorptiometry [DXA]).^(^ ^16^ ^)^ The current gold standard for diagnosing osteoporosis/osteopenia is based on assessing bone mineral density (BMD) at the proximal femur or lumbar spine (LS). These traditional regions of interest (ROIs) are only sometimes optimal for determining the presence of other concurrent tissue loss.

The mid‐thigh ROI has recently been recognized as a clinically relevant and cost‐effective potential site to screen for muscle health by the European Working Group on Sarcopenia in Older People 2 (EWGSOP2).^(^ ^17^ ^)^ Also, there is evidence that tissue masses (bone, lean and fat mass) assessed by DXA scans in the mid‐thigh (2.6 and 13 cm ROIs) are a potential and practical surrogate to study bone, muscle, and fat mass in older people with a previous history of falls and fractures, with comparable ability in predicting muscle performance and falls.^(^ ^18^ ^)^ The aim of this study was to validate the clinical utility of mid‐thigh and whole‐thigh tissue masses in the diagnosis of bone and muscle loss compared to tissue masses in the conventional regions (BMD at LS and hip and appendicular lean mass [ALM]).

## Materials and Methods

### Participants

Based in Geelong, Victoria (Australia), the Geelong Osteoporosis Study is an ongoing population‐based prospective cohort study that has investigated the epidemiology of musculoskeletal conditions since 1993.^(^ ^19^ ^)^ Participants were randomly selected using electoral rolls for the Barwon Statistical Division surrounding Geelong. This study utilized cross‐sectional data and DXA images from the 15‐year follow‐up phase of the study (conducted in 2010–2014 for the female cohort and in 2016–2019 for the male cohort). Complete data were available for 1322 of 1518 participants.

### Measurements

The Geelong Osteoporosis Study collected various clinical, biochemical, questionnaire, and imaging data from participants throughout the study. Specific details can be found in the cohort profile.^(^ ^19^ ^)^ This study included participants' anthropometric measures (weight and height), physical performance measures (timed up‐and‐go, handgrip strength, and gait speed), whole‐body, femur, and spine DXA images, and falls and fractures history questionnaires.

### Image acquisition and analysis

**Fig. 1:**
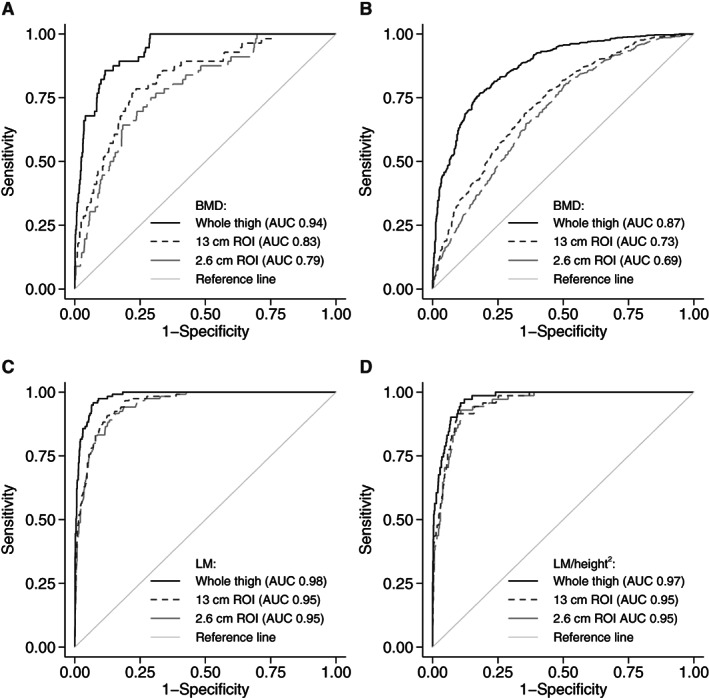
Area under receiver operator characteristics curve (AUC) of thigh ROIs for identifying (*A*) osteoporosis, (*B*) osteopenia using BMD and (*C*) low lean mass – low ALM, (*D*) low ALM/height^2^ using lean mass (LM) and lean mass corrected for height^2^ (LM/heigth^2^) respectively.

Whole‐body, spine, and hip DXA scans of all participants were acquired using a GE Lunar Prodigy machine (GE Medical Systems Lunar, Madison, WI, USA), and BMD and body composition (ALM) were determined using the enCORE software platform (version 14.10.002), as per manufacturer standard protocols with a precision of 0.6% at the LS and 1.6% at the femoral neck (FN). Bone mass of lumbar vertebrae 1 to 4 and the bone mass of FN and total hip (TH) ROI (after visual checks and necessary adjustment) were extracted. All vertebrae were included in the analysis, irrespective of soft tissue artifacts or vertebral compression deformities. Additionally, three new thigh ROIs (three symmetrical pairs) were defined and analyzed on whole‐body scans: 2.6‐ and 13‐cm‐thick left and right mid‐thigh ROI, defined as the midpoint between the upper margin of the greater trochanter of the femur and the lower margin of the medial femoral condyle (Figure 1 in Appendix S1), as well as whole left and right thigh, defined as the region between the lower margin of the ischial tuberosity and the femoral condyles' lower margin. The DXA enCORE software platform estimated bone and lean mass for each region. All ROIs with metallic artifacts or participant positioning issues were excluded from analyses. The average values of right and left ROIs were used for statistical analysis, resulting in three ROIs to be compared with conventional indices: 2.6‐cm mid‐thigh ROI, 13‐cm mid‐thigh ROI, and whole‐thigh ROI. All measures were taken by trained personnel.

### Handgrip strength

Handgrip strength (HGS) was measured using a handheld analog dynamometer (Jamar, Sammons Preston, Bolingbrook, IL, USA) for women and a digital dynamometer (Vernier, LoggerPro3) for men. The testing procedure was demonstrated to participants before the measurement trials. With the participant seated in a comfortable position and the arm holding the dynamometer flexed at the elbow to 90°, the participant was asked to squeeze the device as hard as possible for several seconds, and the peak reading was recorded. This procedure was repeated for each hand. For women, the readings were performed in duplicate on each hand with no time interval between trials, and for men, trials were repeated in triplicate on each hand, holding the peak for 3 seconds with a 5‐second interval between trials. The mean of the maximum value for each hand was used in further analyses. Measures from the Vernier device were transformed to Jamar equivalent values according to the following equation: HGS_Jamar_ (kg) = 9.50 + 0.818 × HGS_Vernier_ (kg) + 8.80 × Sex, where sex = 1 for men, which was developed by measuring the maximum HGS on each device for 45 adults aged 21–67 years.

### Gait assessment

For men only, usual gait speed (m/s) was determined by measuring the time taken (in seconds) to walk a distance of 4 m.

### Falls and fractures

Falls were defined as “when you suddenly find yourself on the ground, without intending to get there, after you were in either a lying, sitting or standing position.”^(^ ^20^, ^21^ ^)^ Self‐reported falls for a year prior to assessment were recorded using a questionnaire. Falls were documented by asking participants (i) whether they had suffered a fall and (ii) the number of falls experienced during the year before the day of the assessment. The occurrence of fractures within the last 5 years (prior to assessment) was evaluated retrospectively and confirmed from radiology clinics in the study region.

### Ethics approval

This study was approved by the Barwon Health Human Research Ethics Committee (HREC Reference Numbers: 92/01_E7 and 00/56_E7). An agreement to collaborate was reached between The University of Melbourne and Barwon Health so as to share the data from the Geelong Osteoporosis Study to be used for analysis (Collaboration Agreement No. TP709555).

### Outcome definition

Osteoporosis and osteopenia were defined as the lowest T‐score from LS, FN, and TH regions using T‐score reference ranges of the gender‐specific young adult population (20–29 years).^(^ ^22^, ^23^ ^)^ These were previously derived using the same GOS database and are recommended by Australia and New Zealand Bone and Mineral Society and Osteoporosis Australia. Low lean mass was defined using EWGSOP2 criteria – ALM <20 kg for men and <15 kg for women; ALM/height^2^ <7.0 kg/m^2^ for men and <5.5 kg/m^2^ for women.^(^ ^17^ ^)^ Low handgrip strength was defined using EWGSOP2 criteria (<27 kg for men and <16 kg for women)^(^ ^17^ ^)^ and using Sarcopenia Definition and Outcomes Consortium (SDOC) criteria (<35.5 kg for men and <20 kg for women).^(^ ^24^ ^)^

### Statistical analysis

All participants with available data were used in the analysis, and no missing data were imputed. Descriptive statistics are presented as median (interquartile range [IQR]) or frequency (percentage). Between‐sex comparisons of baseline characteristics were performed using rank‐sum test and Fisher's exact test. The correlation between thigh ROIs and conventional measures was evaluated using Pearson's correlation coefficient. Area under the receiver operating characteristic curve (AUC) was calculated for each outcome and each ROI separately. AUCs were compared using the algorithm proposed by DeLong, DeLong, and Clarke‐Pearson.^(^ ^25^ ^)^ Optimal cutoffs of BMD and lean mass from thigh ROIs were defined using the Youden index. Sensitivity and specificity with 95% confidence intervals are presented for each suggested cutoff. All analyses were adjusted for sex and were performed using Stata 16.1 (StataCorp LLC, College Station, TX, USA).

## Results

Of a total of 1518 participants, 1321 community‐dwelling participants had available results from DXA scans and are included in the analysis. The median age of included participants was 59 years (IQR 46–70 years), with 38% of the sample being older than 65 years. There was an almost equal split between men and women (43% versus 57%). Additional characteristics of the study population are presented in Table 1.

**Table 1:** Characteristics of Study Population

	Men (*n* = 574)	Women (*n* = 748)	*p* value
Demographics			
Age (years)	64 (51, 73)	55 (42, 67)	<0.001
BMI (kg/m^2^)	27.5 (25.1, 30.1)	27.2 (23.9, 31.8)	0.93
Physical performance			
Timed up and go (s)	8.4 (7.4, 9.7)	7.6 (6.5, 9.0)	<0.001
Handgrip strength (kg)	38.7 (33.3, 43.7)	24 (19.8, 28.5)	<0.001
Gait speed (m/s)	1.1 (0.9, 1.2)	N/A	
Adverse events			
At least 1 fall in last year	83 (14.5%)	201 (26.9%)	<0.001
At least 1 fracture in last 5 years	65 (11.3%)	67 (9.0%)	0.17
BMD (g/cm^2^)			
Neck of femur	0.954 (0.864, 1.055)	0.915 (0.816, 1.021)	<0.001
Hip	1.043 (0.946, 1.148)	0.970 (0.878, 1.069)	<0.001
Lumbar spine	1.298 (1.178, 1.454)	1.205 (1.087, 1.328)	<0.001
2.6 cm mid‐thigh ROI	2.018 (1.874, 2.155)	1.654 (1.490, 1.797)	<0.001
13 cm mid‐thigh ROI	1.986 (1.859, 2.108)	1.624 (1.490, 1.753)	<0.001
Whole thigh ROI	1.608 (1.504, 1.711)	1.393 (1.292, 1.480)	<0.001
Lean mass (kg)			
ALM	25.8 (23.3, 28.4)	17.5 (15.9, 19.3)	<0.001
2.6 cm mid‐thigh ROI	0.42 (0.38, 0.47)	0.31 (0.28, 0.35)	<0.001
13 cm mid‐thigh ROI	2.1 (1.9, 2.4)	1.6 (1.4, 1.7)	<0.001
Whole thigh ROI	5.4 (4.8, 6.0)	3.7 (3.3, 4.1)	<0.001
Lean mass corrected for height^2^ (kg/m^2^)			
ALM	8.4 (7.8, 9.1)	6.7 (6.2, 7.2)	<0.001
2.6 cm mid‐thigh ROI	0.14 (0.13, 0.16)	0.12 (0.11, 0.13)	<0.001
13 cm mid‐thigh ROI	0.69 (0.62, 0.76)	0.60 (0.54, 0.66)	<0.001
Whole thigh ROI	1.8 (1.6, 1.9)	1.4 (1.3, 1.5)	<0.001
Lean mass corrected for BMI (kg/kg/m^2^)			
ALM	0.93 (0.84, 1.05)	0.64 (0.56, 0.72)	<0.001
2.6 cm mid‐thigh ROI	0.015 (0.014, 0.018)	0.011 (0.010, 0.013)	<0.001
13 cm mid‐thigh ROI	0.076 (0.069, 0.085)	0.056 (0.050, 0.063)	<0.001
Whole thigh ROI	0.19 (0.17, 0.22)	0.13 (0.12, 0.15)	<0.001

Continuous variables are presented as median (interquartile range), categorical variables are presented as *n* (%). Between‐group comparison by rank‐sum test and Fisher's exact test.

ALM = appendicular lean mass.

### Clinical utility of thigh regions to identify osteoporosis/osteopenia

The prevalence of osteoporosis was 6.7%, whereas the prevalence of osteopenia was 53.8%. The strongest correlation with conventional BMD was observed with the BMD of whole thigh (*r* = 0.659 for FN, *r* = 0.590 for LS and *r* = 0.745 for TH), whereas BMD in mid‐thigh regions showed weaker correlations (correlation coefficient ranging from 0.374 to 0.494). Whole‐thigh BMD showed an excellent ability to identify osteoporosis (AUC 0.93) and performed better than either the mid‐thigh region (AUC 0.84 for 13 cm ROI and 0.80 for 2.6 cm ROI, *p* < 0.001) (Figure 1*A*). A similar pattern, but with inferior performance, was observed for osteopenia (AUC 0.88 for whole thigh versus 0.73 for 13 cm ROI versus 0.69 for 2.6 cm ROI) (Figure 1*B*). Optimal cutoffs in new ROIs and their associated sensitivities/specificities are presented in Table 2.

**Table 2:** Optimal cutoffs of BMD and lean mass from thigh ROI to identify osteoporosis, osteopenia (both defined by lowest T score from FN, LS, and TH), and low lean mass (defined by ALM or ALM/height^2^ as per EWGSOP2 criteria)

	Proposed cutoff	Sensitivity (95% CI)	Specificity (95% CI)
Osteoporosis			
2.6 cm BMD	1.88 (M), 1.51 (F)	70.5 (59.8, 79.7)	74.9 (72.4, 77.3)
13 cm BMD	1.89 (M), 1.50 (F)	76.1 (65.9, 84.6)	74.9 (72.4, 77.3)
Whole‐thigh BMD	1.51 (M), 1.27 (F)	89.8 (81.5, 95.2)	80.4 (78.0, 82.6)
Osteopenia			
2.6 cm BMD	2.10 (M), 1.70 (F)	75.8 (72.5, 79.0)	53.3 (49.2, 57.3)
13 cm BMD	2.03 (M), 1.68 (F)	76.1 (72.8, 79.2)	57.2 (53.2, 61.2)
Whole‐thigh BMD	1.62 (M), 1.42 (F)	84.3 (81.4, 86.9)	72.7 (69.0, 76.2)
Low lean mass (ALM)			
2.6 cm lean mass	0.35 (M), 0.28 (W)	85.7 (78.1, 91.5)	88.9 (87, 90.7)
13 cm lean mass	1.71 (M), 1.41 (W)	89.9 (83.0, 94.7)	88.7 (86.8, 90.4)
Whole lean mass	4.39 (M) 3.24 (W)	97.5 (92.8, 99.5)	90.9 (89.2, 92.5)
Low lean mass (ALM/height^2^)			
2.6 cm lean mass	0.12 (M), 0.10 (W)	88.7 (79, 95)	90 (88.2, 91.6)
13 cm lean mass	0.59 (M), 0.50 (W)	87.3 (77.3, 94)	85.5 (83.4, 87.4)
Whole lean mass	1.57 (M), 1.18 (W)	91.5 (82.5, 96.8)	91 (89.2, 92.5)

ALM = appendicular lean mass; M = men; W = women.

### Clinical utility of thigh regions to identify low lean mass

Low lean mass was defined by EWGSOP2 criteria using ALM (prevalence 9%) and ALM corrected for height^2^ (5.4% prevalence). There was a high correlation between lean mass in thigh regions and ALM or ALM/height^2^ (*r* > 0.85). All thigh ROIs demonstrated excellent performance in identifying low lean mass (AUC > 0.90), with the whole‐thigh region performing better than mid‐thigh ROIs (*p* < 0.001 for ALM and *p* = 0.022 for ALM/height^2^) (Figure 1*C*,*D*). Suggested cutoffs with high sensitivity and specificity (>85%) in the diagnosis of low lean mass are presented in Table 2.

### Association with low muscle performance and strength

**Fig. 2:**
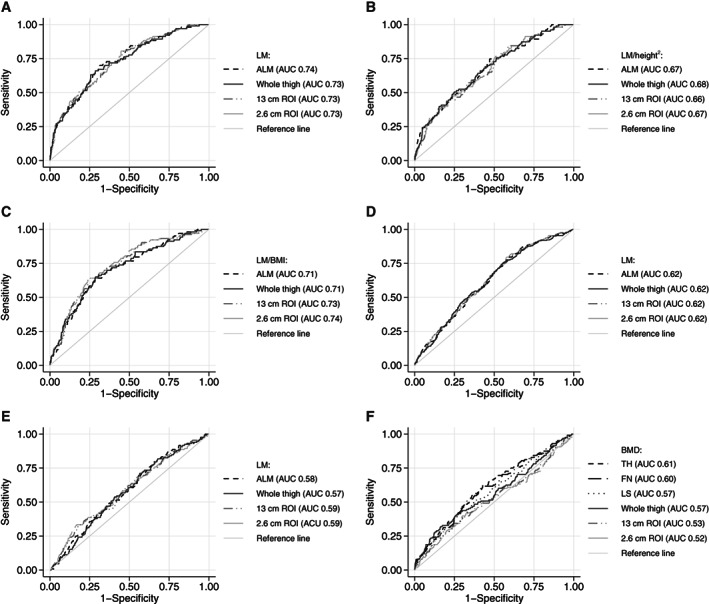
Area under receiver operator characteristic curve (AUC) of thigh ROIs and ALM for identifying low handgrip strength using (*A*) uncorrected lean mass (LM), (*B*) lean mass corrected for heigth^2^, and (*C*) lean mass corrected for BMI; past falls using (*E*) uncorrected lean mass and past fractures using (*F*) uncorrected lean mass and (*G*) BMD.

Low muscle performance and strength were defined as low handgrip strength (as per EWGSOP2 criteria) with a prevalence of 8%. Thigh ROIs were equivalent to ALM in their association with low handgrip strength when they were uncorrected (AUC 0.73; *p* = 0.129) or corrected for height^2^ (AUC 0.67; *p* = 0.208) (Figure 2*A*,*B*). When measures were corrected for BMI, the association with low muscle strength was slightly higher in 2.6‐cm mid‐thigh ROI compared to ALM (AUC 0.74 versus 0.71; *p* = 0.046) (Figure 2*C*). The results of SDOC criteria for low handgrip strength are presented in Table 1 in Appendix S1. Gait speed was measured only in men, and thigh ROIs were equivalent to ALM in their association with low gait speed (when these were uncorrected, AUC 0.60, or corrected for BMI, AUC 0.72). When corrected for height^2^, the whole‐thigh region performed slightly better than ALM (AUC 0.58 versus 0.54; *p* = 0.040) (Figure 2 in Appendix S1).

### Association with past falls and fractures

A total of 21.5% of participants experienced at least one fall a year prior to the assessment, and 10% had experienced at least one fracture 5 years prior. ALM and lean mass from thigh regions were equally poor in their association with past falls (AUC = 0.62) and past fractures (AUC <0.60) (Figure 2*D*,*E*). Similarly, BMD from conventional regions performed poorly in their association with past fractures (AUC ≤0.61), and all thigh regions performed worse (compared to FN and TH, but not LS, AUC <0.57) (Figure 2*F*).

## Discussion

In a large population of community‐dwelling adults, thigh regions (especially the whole‐thigh region) show a good ability to identify low bone mass (particularly osteoporosis with sensitivity above 80%) and low lean mass (sensitivity above 85%). Their performance in identifying low muscle strength was equivalent to that of the conventional indices. Lean mass in thigh regions showed equivalent associations to past falls and fractures as ALM, whereas mid‐thigh BMD performed worse than FN or TH BMD.

The rationale for focusing our analyses on the mid‐thigh region by DXA comes from previous clinical evidence. A recent pilot study reported that the mid‐thigh has significant potential in assessing bone, lean, and fat mass in a “one‐stop” DXA scan within a fraction of time, expected lower radiation, and easier positioning than those involved in conventional scans,^(^ ^18^ ^)^ conditions that would be particularly advantageous in challenging settings such as ICUs, emergency departments, or residential aged care facilities where a whole body DXA scan is challenging or burdensome. In addition, the mid‐thigh ROI has recently been recognized as a potential site to screen for sarcopenia by EWGSOP2 that is clinically relevant and cost‐effective.^(^ ^17^ ^)^ Although sarcopenia definitions mostly utilize muscle strength measures,^(^ ^17^, ^24^ ^)^ DXA‐derived lean mass is still valuable in circumstances where the patient's physical or cognitive impairment prevents accurate assessment of muscle mass with functional tests.

The mid‐thigh region contains the largest muscle volume in the body and a considerable amount of cortical bone.^(^ ^1^ ^)^ It has been shown that DXA‐derived mid‐thigh lean mass strongly correlates with cross‐sectional muscle area determined by CT of the same region.^(^ ^26^ ^)^ Furthermore, estimates of skeletal muscle mass determined from mid‐thigh ROI by MRI scan are less variable and have a higher correlation with whole‐body muscle mass than other ROIs.^(^ ^27^ ^)^ This could be partly due to the lack of ligaments or relatively voluminous tendons in the mid‐thigh region and, considering the similar X‐ray attenuation constants between muscles, tendons, and ligaments, lean and muscle mass can be measured with greater accuracy using DXA or MRI, respectively. Regarding bone, although the mid‐thigh is mainly composed of cortical bone, there is evidence that low cortical bone structure combined with a small muscular area in the mid‐thigh is associated with high fracture risk in both sexes.^(^ ^28^ ^)^

Our results also resonate with those of Zemel et al.,^(^ ^29^ ^)^ who showed that measuring distal, mid‐distal, and mid‐femoral BMD of those under 18 years (acquired in lateral projection) is valid for clinical practice due to the significant associations between BMD in this region and clinical outcomes, including response to bisphosphonate therapy and fractures in children. Indeed, the International Society for Clinical Densitometry adopted this ROI as an acceptable measure of BMD in children.^(^ ^30^ ^)^ The convenience and usefulness of scanning the region in children, particularly those with spinal abnormalities, cerebral palsy, or acute immobilization, is another benefit of the technique that resonates with geriatrics practice.

Although the principles of DXA technology are widely available and understood, current machines cannot be brought to challenging settings as described earlier; therefore, technology adaptation would be required to implement regular assessment of mid‐thigh DXA scanning in clinical practice. DXA scans focusing on a smaller ROI and higher portability would be expected to assess the mid‐thigh region in a few seconds up to a minute, which can also be done efficiently in a sitting position. This would decrease operational costs and make the technology even more affordable and accessible than CT and MRI, with the former being subject to high radiation exposure. The short duration of mid‐thigh DXA is particularly ideal for those who find lying flat difficult or uncomfortable due to conditions such as back pain, severe spinal abnormalities, vertebral fractures, heart failure, chronic lung disease, and paroxysmal positional vertigo, which are common in older patients. Additionally, being away from joints, the mid‐thigh is not directly affected by periarticular changes such as osteoarthritic sclerosis (e.g., hip), osteophytes or spondylosis, or vertebral compression fractures. Also, due to the relatively round geometry of the mid‐thigh muscles and bone and fat distribution, no particular positioning (such as pronation of the leg to make femoral neck perpendicular to the radiation axis) is required for this region (unlike mid‐calf, forearm, or femoral neck/hip scans).

Overall, considering the aforementioned advantages, standardization of bone, muscle, and fat mass in the mid‐thigh area and determination of T‐ and Z‐scores for tissue masses in that ROI can lead to a quick, low‐cost, and potentially even lower‐radiation diagnostic tool for various body composition syndromes exemplified earlier. Although tissue masses in both 2.6‐ and 13‐cm mid‐thigh ROI could be used, a wider ROI (whole thigh) performs better due to a lower chance of random error.

A strength of the study is the large random sample of the population, which aided in the generalizability of the results. DXA scans and assessments were performed concurrently, and data were collected prospectively. Reported fractures were radiographically confirmed; however, the major limitation is the retrospective collection of falls and fractures, which prevented us from assessing the predictive ability of these new ROIs. Gait speed was collected only for the male population, and no other longitudinal assessments were available. No other external data were available to validate the proposed cutoffs.

In conclusion, mid‐thigh and whole‐thigh lean and bone mass can be used for diagnosing bone and muscle loss and performed equivalently to conventional measures in diagnosing low muscle strength and function. These results warrant further longitudinal research to explore age‐associated changes in bone, muscle, and fat mass and the predictive value of this ROI for adverse outcomes (i.e., falls and fractures). Adapting the technology to scan the mid‐thigh may create a low‐cost, low‐radiation, and fast screening tool that can facilitate the implementation of DXA scanning protocols for various conditions and settings (e.g., ICUs, nursing homes).

## Author Contributions

**Sara Vogrin:** Conceptualization; data curation; formal analysis; methodology; writing – original draft; writing – review and editing. **Ebrahim Bani Hassan:** Conceptualization; data curation; investigation; methodology; writing – original draft. **Fernan Munandar Putra:** Data curation; investigation; writing – original draft; writing – review and editing. **Julie A Pasco:** Conceptualization; data curation; formal analysis; investigation; methodology; supervision; writing – original draft; writing – review and editing. **Mark A Kotowicz:** Data curation; funding acquisition; investigation; methodology; supervision; writing – original draft; writing – review and editing. **Gustavo Duque:** Conceptualization; data curation; funding acquisition; investigation; methodology; project administration; supervision; writing – original draft; writing – review and editing.

## Conflict of Interest

SV, EBH, FMP, JAP, MAK, and GD have no conflicts to declare.

## Supporting information


**Appendix S1.** Supporting Information

## Data Availability

Data sharing is not applicable to this article as no new data were created in this study.

## References

[1] Gingrich A , Volkert D , Kiesswetter E , et al. Prevalence and overlap of sarcopenia, frailty, cachexia and malnutrition in older medical inpatients. BMC Geriatr. 2019;19(1):120. 31029082 10.1186/s12877-019-1115-1 PMC6487020

[2] Petermann‐Rocha F , Pell JP , Celis‐Morales C , Ho FK . Frailty, sarcopenia, cachexia and malnutrition as comorbid conditions and their associations with mortality: a prospective study from UK biobank. J Public Health. 2021;44:e172‐e180. 10.1093/pubmed/fdaa226 PMC9234318 33423060

[3] Crow RS , Lohman MC , Titus AJ , et al. Association of Obesity and Frailty in older adults: NHANES 1999‐2004. J Nutr Health Aging. 2019;23(2):138‐144. 30697622 10.1007/s12603-018-1138-x PMC6371801

[4] Hirschfeld HP , Kinsella R , Duque G . Osteosarcopenia: where bone, muscle, and fat collide. Osteoporos Int. 2017;28(10):2781‐2790. 28733716 10.1007/s00198-017-4151-8

[5] Hamrick MW , McGee‐Lawrence ME , Frechette DM . Fatty infiltration of skeletal muscle: mechanisms and comparisons with bone marrow adiposity. Front Endocrinol. 2016;7:69. 10.3389/fendo.2016.00069 PMC4913107 27379021

[6] Shen Y , Chen J , Chen X , Hou L , Lin X , Yang M . Prevalence and associated factors of sarcopenia in nursing home residents: a systematic review and meta‐analysis. J Am Med Dir Assoc. 2019;20(1):5‐13. 30409494 10.1016/j.jamda.2018.09.012

[7] Pasco J , Holloway‐Kew K , Hyde N , et al. Pretiree lifestyles in relation to musculoskeletal health: cross‐sectional data from the Geelong osteoporosis study. JCSM Clin Rep. 2018;3:3.

[8] Greco EA , Pietschmann P , Migliaccio S . Osteoporosis and sarcopenia increase frailty syndrome in the elderly. Front Endocrinol. 2019;10:255. 10.3389/fendo.2019.00255 PMC6491670 31068903

[9] Parry SM , Puthucheary ZA . The impact of extended bed rest on the musculoskeletal system in the critical care environment. Extrem Physiol Med. 2015;4:16. 26457181 10.1186/s13728-015-0036-7 PMC4600281

[10] Maimoun L , Garnero P , Mura T , et al. Specific effects of anorexia nervosa and obesity on bone mineral density and bone turnover in young women. J Clin Endocrinol Metab. 2020;105(4):dgz259. 31821467 10.1210/clinem/dgz259

[11] Peixoto da Silva S , Santos JMO , Costa ESMP , Gil da Costa RM , Medeiros R . Cancer cachexia and its pathophysiology: links with sarcopenia, anorexia and asthenia. J Cachexia Sarcopenia Muscle. 2020;11(3):619‐635. 32142217 10.1002/jcsm.12528 PMC7296264

[12] Sepulveda‐Loyola W , Osadnik C , Phu S , Morita AA , Duque G , Probst VS . Diagnosis, prevalence, and clinical impact of sarcopenia in COPD: a systematic review and meta‐analysis. J Cachexia Sarcopenia Muscle. 2020;11(5):1164‐1176. 32862514 10.1002/jcsm.12600 PMC7567149

[13] Fukuta A , Saito T , Murata S , et al. Impact of preoperative cachexia on postoperative length of stay in elderly patients with gastrointestinal cancer. Nutrition. 2019;58:65‐68. 30316109 10.1016/j.nut.2018.06.022

[14] Chang CD , Wu JS , Mhuircheartaigh JN , et al. Effect of sarcopenia on clinical and surgical outcome in elderly patients with proximal femur fractures. Skeletal Radiol. 2018;47(6):771‐777. 29247259 10.1007/s00256-017-2848-6

[15] Sanchez‐Rodriguez D , Annweiler C , Ronquillo‐Moreno N , et al. Prognostic value of the ESPEN consensus and guidelines for malnutrition: prediction of post‐discharge clinical outcomes in older inpatients. Nutr Clin Pract. 2019;34(2):304‐312. 30070732 10.1002/ncp.10088

[16] Bauer J , Morley JE , Schols A , et al. Sarcopenia: a time for action. An SCWD position paper. J Cachexia Sarcopenia Muscle. 2019;10(5):956‐961. 31523937 10.1002/jcsm.12483 PMC6818450

[17] Cruz‐Jentoft AJ , Bahat G , Bauer J , et al. Sarcopenia: revised European consensus on definition and diagnosis. Age Ageing. 2019;48(4):601. 10.1093/ageing/afz046 PMC6593317 31081853

[18] Bani Hassan E , Phu S , Vogrin S , et al. Diagnostic value of mid‐thigh and mid‐calf bone, muscle, and fat mass in Osteosarcopenia: a pilot study. Calcif Tissue Int. 2019;105(4):392‐402. 31292687 10.1007/s00223-019-00582-5

[19] Pasco JA , Nicholson GC , Kotowicz MA . Cohort profile: Geelong osteoporosis study. Int J Epidemiol. 2012;41(6):1565‐1575. 23283714 10.1093/ije/dyr148

[20] Cwikel JG , Fried AV , Biderman A , Galinsky D . Validation of a fall‐risk screening test, the elderly fall screening test (EFST), for community‐dwelling elderly. Disabil Rehabil. 1998;20(5):161‐167. 9622261 10.3109/09638289809166077

[21] Lamb SE , Jorstad‐Stein EC , Hauer K , Becker C . Prevention of falls network E, outcomes consensus G. development of a common outcome data set for fall injury prevention trials: the prevention of falls network Europe consensus. J Am Geriatr Soc. 2005;53(9):1618‐1622. 16137297 10.1111/j.1532-5415.2005.53455.x

[22] Henry MJ , Pasco JA , Korn S , Gibson JE , Kotowicz MA , Nicholson GC . Bone mineral density reference ranges for Australian men: Geelong osteoporosis study. Osteoporos Int. 2010;21(6):909‐917. 19707703 10.1007/s00198-009-1042-7

[23] Henry MJ , Pasco JA , Pocock NA , Nicholson GC , Kotowicz MA . Reference ranges for bone densitometers adopted Australia‐wide: Geelong osteoporosis study. Australas Radiol. 2004;48(4):473‐475. 15601326 10.1111/j.1440-1673.2004.01351.x

[24] Bhasin S , Travison TG , Manini TM , et al. Sarcopenia definition: the position statements of the sarcopenia definition and outcomes consortium. J Am Geriatr Soc. 2020;68(7):1410‐1418. 32150289 10.1111/jgs.16372 PMC12132920

[25] DeLong ER , DeLong DM , Clarke‐Pearson DL . Comparing the areas under two or more correlated receiver operating characteristic curves: a nonparametric approach. Biometrics. 1988;44(3):837‐845. 3203132

[26] Hansen RD , Williamson DA , Finnegan TP , et al. Estimation of thigh muscle cross‐sectional area by dual‐energy X‐ray absorptiometry in frail elderly patients. Am J Clin Nutr. 2007;86(4):952‐958. 17921370 10.1093/ajcn/86.4.952

[27] Lee SJ , Janssen I , Helymsfield SB , Ross R . Relation between whole‐body and regional measures of human skeletal muscle. Am J Clin Nutr. 2004;80(5):1215‐1221. 15531668 10.1093/ajcn/80.5.1215

[28] Johannesdottir F , Aspelund T , Siggeirsdottir K , et al. Mid‐thigh cortical bone structural parameters, muscle mass and strength, and association with lower limb fractures in older men and women (AGES‐Reykjavik study). Calcif Tissue Int. 2012;90(5):354‐364. 22451219 10.1007/s00223-012-9585-6 PMC5111551

[29] Zemel BS , Stallings VA , Leonard MB , et al. Revised pediatric reference data for the lateral distal femur measured by Hologic discovery/Delphi dual‐energy X‐ray absorptiometry. J Clin Densitom. 2009;12(2):207‐218. 19321369 10.1016/j.jocd.2009.01.005 PMC4641747

[30] Weber DR , Boyce A , Gordon C , et al. The utility of DXA assessment at the forearm, proximal femur, and lateral distal femur, and vertebral fracture assessment in the pediatric population: 2019 ISCD official position. J Clin Densitom. 2019;22(4):567‐589. 31421951 10.1016/j.jocd.2019.07.002 PMC7010480

