# Supplementary material for: Clinical Utility of Thigh and Mid‐Thigh Dual‐Energy x‐Ray Absorptiometry to Identify Bone and Muscle Loss
Source: JBMR Plus. 2023 Feb 16;7(4):e10704. doi: 10.1002/jbm4.10704 (PMC10097639; doi:10.1002/jbm4.10704)
Supplement: Supplementary file 1 — Appendix S1. Supporting Information [file JBM4-7-e10704-s001.docx]

**Supplemental material**

***Supplemental Figure 1.*** *Three pairs (six) ROIs were defined and analyzed on whole-body scans. The average of right and left ROIs was used for analyses: 2.6 cm thick mid-thigh ROI is presented in purple rectangle (ROI 1 and 2), 13 cm thick mid-thigh ROI is presented in blue rectangle (ROI 3 and 4). Both mid-thigh ROIs were defined as the mid-point between the upper margin of the greater trochanter of the femur and the lower margin of the medial femoral condyle. Whole thigh ROI is presented in red rectangle (ROI 5 and 6) and was defined as the region between the lower margin of the ischial tuberosity and the lower margin of the femoral condyles.*


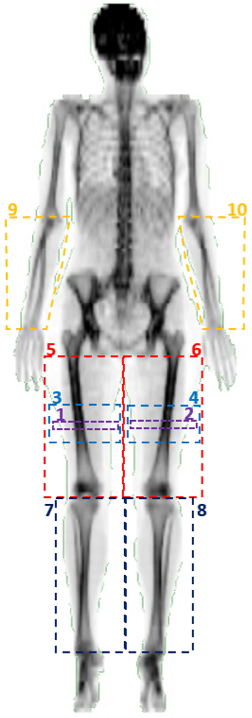


***Supplemental Figure 2.*** *Area under the receiver operator characteristics curve (AUC) of thigh ROIs and appendicular lean mass (ALM) for identifying* ***low gait speed*** *in male sample using uncorrected lean mass (LM) (A), lean mass corrected for BMI (B) and lean mass corrected for height^2^ (C).*

**

***Supplemental Table 1****: Area under the receiver operator characteristics curve (AUC) of lean mass from thigh ROIs and appendicular lean mass (ALM) for diagnosis low handgrip strength using SDOC criteria.*

|  | **Uncorrected** | | **Corrected for BMI** | | **Corrected for height^2^** | |
| --- | --- | --- | --- | --- | --- | --- |
|  | AUC (95% CI) | P value | AUC (95% CI) | P value | AUC (95% CI) | P value |
| **ALM** | 0.73 (0.70, 0.76) | 0.206 | 0.75 (0.72, 0.78) | 0.088 | 0.65 (0.62, 0.68) | <0.001 |
| **13 cm ROI** | 0.71 (0.68, 0.74) |  | 0.76 (0.74, 0.79) |  | 0.62 (0.58, 0.65)^a^ |  |
| **2.6 cm ROI** | 0.72 (0.69, 0.75) |  | 0.77 (0.74, 0.80) |  | 0.62 (0.59, 0.66)^b^ |  |
| **Whole thigh** | 0.72 (0.69, 0.75) |  | 0.75 (0.72, 0.78) |  | 0.66 (0.62, 0.69) |  |

AUC=area under the receiver operator characteristics curve; ALM=appendicular lean mass;

^a^p<0.001 when compared to ALM; ^b^p=0.003 when compared to ALM
